# The relation between Self-Esteem and Productivity: An analysis in higher education institutions

**DOI:** 10.3389/fpsyg.2022.1112437

**Published:** 2023-01-11

**Authors:** Fabiola Gómez-Jorge, Eloísa Díaz-Garrido

**Affiliations:** Business Organization, Department of Business Economics, Rey Juan Carlos University, Madrid, Spain

**Keywords:** Self-Esteem, work environment, Productivity, education, sustainability

## Abstract

**Background:**

Due to the importance of academic training, allowing upward socioeconomic mobility, and being key to getting out of poverty, as indicated by the United Nations in its 2030 Agenda, investment in quality education is key. The objective of this study is to analyze the impact of Self-Esteem in the work environment on Teaching and Research Productivity within the field of higher education in Spain.

**Method:**

The research is carried out among the teaching staff of the Rey Juan Carlos University of Madrid (Spain). A structured questionnaire was used to ask about Self-Esteem and Productivity. Data analysis is conducted using 272 valid questionnaires analyzed with R-commander software. The validity of the variables is analyzed to check the quality of the questionnaire. Linear regression analysis is used to examine the relationship between Self-Esteem and Productivity and is completed with ANOVA analysis to study the most significant differences between these variables.

**Results:**

We identified a positive correlation between Self-Esteem and Productivity, where significant differences have been observed depending on the age and seniority of the teaching staff.

**Conclusion:**

This research contributes positively to the achievement of Sustainable Development Goals 3 (SDG3) (Good Health and Wellbeing) and 4 (Quality Education), in addition to highlighting the importance of universities ensuring the Self-Esteem of their teachers, having a very positive impact on the education received by the students, on the quality and prestige of the teaching center, and society, increasing academic research and educational quality. Similarly, the results achieved can be extrapolated to other sectors.

## 1. Introduction

The evidence that happy workers are more productive and deal more effectively with high workplace expectations has fostered the tendency of organizations to increase their concern for the health and wellbeing of their employees (Lyubomirsky et al., 2005; Zelenski et al., 2008; Pfeffer, 2018; Tonkin et al., 2018).

Self-Esteem has a significant impact on many essential results of employees, being identified as a determining variable on their behavior both inside and outside the workplace, affecting performance, satisfaction, commitment, turnover, work motivation, and even on the civic behavior of workers (Campbell, 1990; Pierce and Gardner, 2004).

In the specialized literature, we find studies on Self-Esteem in the work environment in areas such as the hospitality industry (Wang et al., 2020; Kim et al., 2021), construction (Wu et al., 2019), the pharmaceutical industry (Costantini et al., 2019), high technology (Norman et al., 2015), manufacturing (Elloy, 2005; Chan et al., 2012; Pan et al., 2014), banking (Lee, 2003; Lee and Peccei, 2007; Liu et al., 2013), mining (Pierce and Gardner, 2009), or the electricity sector (Tharenou and Harker, 1982).

Studies have been observed within the higher field that analyze the relationship between Self-Esteem and academic results from the student’s point of view (Chilca Alva, 2017; Shin, 2018). However, no works have been identified that analyze the direct impact of Self-Esteem on the Productivity of workers within the field of Higher Education, only authors such as Takhsha et al. (2020) or Shabeer et al. (2020) have analyzed Self-Esteem in the work environment as a moderating variable of supervisor leadership, career adaptability of subordinates and ostracism within their studies at the university level.

Due to the importance of academic training, allowing upward socioeconomic mobility, and being key to getting out of poverty, as indicated by the United Nations in its 2030 Agenda, investment in quality education is key. Through this research, it is intended to contribute to the achievement of Sustainable Development Goals 4 (SDG4): Quality Education and SDG 3: Good Health and Wellbeing. This study will allow us to propose important contributions at a practical level very useful for universities, evidencing the impact that teachers’ Self-Esteem has on their labor Productivity, far-reaching for students, higher education centers, and society.

Thus, the objective of this study is to analyze the impact of Self-Esteem in the work environment on the Productivity of teachers within the field of higher education in Spain. Throughout the study, a distinction is made between Teaching and Research Productivity, as these are the two main functions held by university teaching staff.

The empirical study was carried out at the Rey Juan Carlos University (URJC) in Madrid. Specifically, teachers at this university were surveyed and a total of 272 valid questionnaires were obtained. The results of the statistical analysis show a positive relationship between the Self-Esteem of university teachers and their Teaching and Research Productivity. Although significant differences have been identified according to age and professional category of the teaching staff. Within the URJC, there are organizational units (Healthy University) that develop programs in order to promote the construction of personal skills that help people feel better and function optimally in their day to day. The present study will make contributions in this regard. The originality of this article lies in the fact that the results of the study will allow the development of action plans with the aim of improving the Self-Esteem and Productivity of employees within their scope of work.

This article is divided into four sections. The first section offers a small review of the literature, the hypothesis statement is exposed, and the research model is defined. In the second, the methodology used is described. Next, in the third section, the results achieved are developed. Finally, in the fourth part, the conclusion of the study, the limitations, and future lines of research are suggested.

## 2. Literature review and hypothesis approach

Organization-Based Self-Esteem refers to the perceived value that an employee experiences about himself as a result of his participation in an organization and reflects whether that person feels valued and recognized as a competent and effective individual in such a context. Workers who have a high level of Self-Esteem are perceived as capable, irreplaceable, significant, competent, and as playing a valuable role in the organization (Pierce et al., 1989; Pierce and Gardner, 2004; Kim and Beehr, 2018; Neves et al., 2020; Rice et al., 2020).

The level of Organization-Based Self-Esteem is determined by factors such as individual Self-Esteem, experiences within the organization, or its structure and values. Self-Esteem in the workplace is socially determined, being shaped through interactions with others, the social learning experience, and relating to received and internalized social messages from significant others (Brookover et al., 1964; Korman, 1970; Brockner, 1988; Baumeister, 1999; McAllister and Bigley, 2002; Pierce and Gardner, 2004; Kim and Beehr, 2018).

The work experience is affected by emotions and state of mind. Individuals’ reaction to work events varies over time and drives their immediate affective states. In addition, it should be noted how a positive state of mind increases the probability of the occurrence of positive events and vice versa (Weiss and Cropanzano, 1996), hence, the importance of ensuring the state of mind of workers.

Self-Esteem has a significant impact on many essential employee outcomes, such as motivation, attitudes (job satisfaction, turnover intention, or organizational commitment), behavior (civic behavior), or overall job performance (Campbell, 1990; Pierce and Gardner, 2004). As people dedicate a large part of their lives to work, Self-Esteem in the work environment will play an important and significant role in the total scheme of their lives (Gardner and Pierce, 2011).

### 2.1. Self-Esteem and Productivity

In the specialized literature, both positive and negative relationships established between Organization-Based Self-Esteem and its impact on effectiveness are frequently analyzed. Efficacy, understood within the work context, is considered to improve with the increase in Organization-Based Self-Esteem (Pan et al., 2014), since employees with high Self-Esteem engage in learning behaviors more frequently than those who have a low level of Self-Esteem, since they avoid participating in the organization for fear of failure, thus missing out on opportunities for success (Hahn and Mathews, 2018; Lin et al., 2018).

Regarding performance, there are numerous papers that show how Self-Esteem helps employees to improve their performance, helping them to cope with work stress, anxiety, and depression. In addition, this performance is related to the Productivity of employees and the possible impact on the progression of their professional careers (Tharenou, 1979; Gardner et al., 2004; Brough et al., 2009; Gordon and Hood, 2020; Kim et al., 2021). Although the research by Zelenski et al. (2008) suggests that happiness can stimulate productivity.

The fact that a quarter of job performance is explained by positive wellbeing is very illuminating since positive wellbeing acts as a significant and positive predictor of employee performance (Luna-Arocas and Danvila-del-valle, 2020).

In general, individuals with a high level of Self-Esteem (in addition to other variables such as internal locus of control, generalized self-efficacy, and emotional stability) tend to be happier at work, as well as in other areas of life. In addition, individuals present higher levels of happiness when they consider that their performance is better than usual (Fisher, 2010).

There are prominent differences in the attitudes that employees present according to their level of Self-Esteem, such as perceiving certain situations at work as a challenge or an opportunity, for employees with high Self-Esteem, or identifying the same situation as a threat, for workers with low Self-Esteem. For those individuals who have weakened Self-Esteem, higher levels of social anxiety, need for approval, and sensitivity to evaluations made by third parties have been identified, in addition to having a greater probability of experiencing emotional dissonance (Schuler, 1980; Abraham, 1999; Vermunt and Steensma, 2001).

In addition, factors such as job stress, ambiguity, conflict or role overload, and job insecurity are negatively associated with Self-Esteem in the work environment due to the potential it takes to positively disrupt successful job performance. Other situations such as unemployment, for example, have been linked to feelings of depression by damaging a person’s Self-Esteem (Goldsmith et al., 1996; Bowling, 2011).

Employees and organizations can be affected by the health and wellbeing of their workers since the presence of these problems result in lower productivity, lower quality decisions, higher absenteeism, and lower contributions to the organization (Danna and Griffin, 1999).

The reduction of the workload and the stressors of work, the increase in the complexity and autonomy of the position, job achievement, support for workers and their empowerment, or practices such as mentoring, are identified as variables that help to improve efficacy and work Self-Esteem (Pierce and Gardner, 2004; Lee and Peccei, 2007; Ferris et al., 2009; Singh et al., 2009; Bowling et al., 2010; Gardner and Pierce, 2013; Liu et al., 2013; Lin et al., 2018; Wu et al., 2019).

Research that includes the influence of Self-Esteem on work efficiency has been carried out in the health sector (Carson et al., 1997), pharmacist (Costantini et al., 2019), food sector (Fadilah et al., 2018), hotelier (Lin et al., 2018), banking (Liu et al., 2013), mining (Gardner and Pierce, 2011), construction (Wu et al., 2019), and in the high-tech industry (Norman et al., 2015). An investigation has been carried out in Indonesia (Fadilah et al., 2018), Korea (Hahn and Mathews, 2018), China (Liu et al., 2013; Lin et al., 2018; Yang et al., 2018), and the United States (Gardner and Pierce, 2011).

Moreover, the studies carried out by Shabeer et al. (2020) and Takhsha et al. (2020) were conducted at higher education centers in Pakistan and Iran, respectively. In them, they only identified Self-Esteem in the work environment as a moderating variable of supervisor leadership, career adaptability of subordinates, and ostracism. Therefore, no specific investigations have been identified about the impact of Self-Esteem on Productivity. Studies carried out in Europe, specifically in Spain, or in the field of higher education have not been identified either.

Based on the premise that the increase in Organization-Based Self-Esteem improves individual efficacy (Pan et al., 2014), the following hypothesis is proposed:

*Hypothesis 1. Self-Esteem improves worker Productivity.*

## 3. Methodology

The information necessary to undertake the empirical study has been obtained through a structured questionnaire, given the impossibility of obtaining this data from secondary sources. Different phases were carried out for the elaboration of the questionnaire. First, the specialized literature was reviewed in order to identify the measures for each of the variables that make up the analysis model. Second, a pre-test was carried out with the aim of improving the initial questionnaire. For this, personal interviews were arranged with five academics. The object of these interviews was the analysis of the facility to answer the questionnaire and understand it. This made it possible to introduce improvements and modify certain questions that were not easy to answer as they were formulated.

The final questionnaire is made up of a total of 18 questions designed to assess the variables of the analysis model, that is, Self-Esteem and Productivity.

To measure Self-Esteem, the Rosenberg Self-Esteem Scale (Rosenberg, 1965) has been used, validated by this same author in 1965 and by Atienza et al. (2000). In order to analyze Self-Esteem in the work environment, the questions are adapted to the work environment. The questionnaire consists of 10 items, with five positively described sentences (e.g., “I am convinced that I have good qualities to perform my job”) and five negatively (e.g., “I feel that I do not have much of what to be proud of in my workplace”). The possible answers are framed on a Likert scale of five options (“strongly disagree,” “disagree,” “neither agree nor disagree,” “agree,” and “strongly agree”). In Supplementary Annex 1, it is possible to observe the items used.

To measure the Productivity variable, a distinction has been made between the Teaching Productivity and Research Productivity of the respondents, considering that university teachers, within their position, must perform both tasks (teaching and research) as established in the Organic Law of Universities (Boletín Oficial del Estado (2001) LOU, by its Spanish acronym), in the first article of the Preliminary Title in its first section establishes that the function of the University is to carry out the public service of higher education through research, teaching, and study. To measure Teaching Productivity, the item “Teacher Evaluation” is used, where the result obtained in the Teacher Evaluation Surveys conducted by the University and answered by the students is taken as a reference (1 being the minimum level and 5 being the maximum level). It offers a Likert scale with four response options (“less than 2,” “between 2 and 3,” “between 3 and 4,” and “greater than 4”).

To measure Research Productivity, the items “Publications”, “Annual conferences”, and “Research projects” are taken, thus considering the merits at the scientific-academic level included in the principles and guidelines for the evaluation criteria of the National Agency for Quality Assessment and Accreditation research (ANECA, by its Spanish acronym). Therefore, four questions are posed with the objective of knowing the total number of Publications carried out throughout the academic career by the participants, the number of Conferences they usually attend annually, and the number of Research Projects or contracts throughout their careers in which they have served as both principal investigators and team members. Supplementary Annex 2 includes the questions used to measure Productivity.

The sample is made up of a group of teachers belonging to Rey Juan Carlos University (Madrid, Spain). The response rate is within acceptable limits since the 272 valid questionnaires they are fully representative of the total population because it can be stated that with a confidence level of 95% the margin of error would be 0.0032.

Sample error $\sqrt{\frac{\left(N-n\right)}{n\,\left(N-1\right)}}$, *where N is the population and n the available sample.*

## 4. Results

Regarding the analysis of the validity of the measures used to measure the Self-Esteem and Productivity variables, it stands out that the validity of the content has been ensured thanks to the process of elaboration and revision of the questionnaire. Specifically, the set of representative items of the Self-Esteem variable comes from investigations where good reliability and validity results have been achieved, specifically Rosenberg (1965) and Atienza et al. (2000), and have been adapted to the characteristics specific to this research. With regard to the Productivity variable, the items representative of the merits that are considered when evaluating the activity of university teaching staff by the assessment and accreditation agencies of teaching staff activity have been included, such as ANECA (National Agency for Quality Assessment and Accreditation), in the field of Spanish Universities. In addition, the validity of the content is completed thanks to the review, critique, and pre-test of the initially proposed questionnaire.

First, we proceed to describe and analyze the sample of teachers who have responded to the survey (Table 1). Regarding gender, the sample is made up of 45.96% of men and 54.04% of women, which shows a certain homogeneity in terms of the gender of the respondents.

**TABLE 1:** Distribution of the sample according to gender, age, seniority, and professional category.

	Freq.
**Gender**	
Male	45.96%
Female	54.04%
**Age**	
Less than 30 years old	7.35%
Between 30 and 40 years old	32.35%
Between 40 and 50 years old	26.47%
Between 50 and 60 years old	27.94%
More than 60 years old	5.88%
**Seniority**	
Less than 5 years	21.32%
Between 5 and 10 years	26.84%
Between 10 and 15 years	13.60%
Between 15 and 20 years	14.71%
More than 20 years	23.53%
**Professional category**	
Professor	6.99%
Interim Senior Lecturer	1.10%
Senior Lecturer	23.16%
Interim Tenured	0.37%
Interim Contracted Lecturer	6.99%
Contracted Lecturer	7.35%
Assistant Lecturer	11.40%
Research Assistant	2.94%
Visiting Lecturer	21.32%
Associate Lecturer	18.38%

Regarding age, the most represented teachers are between 30 and 40 years old (32.35%), followed by those between 50 and 60 (27.94%), and between 40 and 50 years old (26.47%), respectively. The least represented are those over 60 years old (5.88%) and those under 30 (7.35%). Thus, more than half of the sample is between the ages of 30 and 50 years old.

Referring to seniority, 26.84% of the sample has seniority in the university field of between 5 and 10 years, being the most represented. Followed are those with an age of more than 20 years (23.53%) and less than 5 years (21.32%). The least represented are those who have been working between 15 and 20 years in the university environment (14.71%) and between 10 and 15 years (13.60%). It should be noted that more than half of those surveyed have been at the university for less than 20 years.

With respect to the professional category, 23.16% hold the position of Senior Lecturer, with the highest level of representation. Next, are the Visiting Lecturer (21.32%), Associate Lecturer (18.38%), and Assistant Lecturer (11.40%). On the contrary, the professional categories less present in the sample are Interim Tenured (0.37%), Interim Senior Lecturer (1.10%), Research Assistant (2.94%), Professor (6.99%), Interim Contracted Lecturer (6.99%), and Contracted Lecturer (7.35%). These results are consistent and correspond to percentages similar to the total URJC teaching staff in each category, which shows that the available sample can be considered sufficiently representative to carry out rigorous statistical analysis.

Second, the basic descriptive statistics for the variables analyzed are presented (Table 2). Regarding the explanatory variable Self-Esteem, and according to the Rosenberg Self-Esteem Scale (Rosenberg, 1965), respondents obtain three possible results: high, medium, or low Self-Esteem. We assigned the following values to these results: high Self-Esteem (3), intermediate Self-Esteem (2), and low Self-Esteem (1). The average of the explanatory variable Self-Esteem of all the respondents is 2.73, that is, close to the high level (3) and below the intermediate level (2). Therefore, an intermediate-high Self-Esteem is observed among the respondents.

**TABLE 2:** Descriptive statistics of the variables.

		Mean	Standard deviation	cv
	Self-Esteem	2.73	0.59	0.22
Research Productivity	Annual Conferences	1.70	1.78	1.04
	Research Projects	8.75	10.96	1.25
	Publications	27.94	38.17	1.36
Teaching Productivity	Teacher Evaluation	3.63	0.60	0.16

To study the variables explained, Teaching Productivity, the item Teacher Evaluation is taken as a reference, while for Research Productivity, the items Annual Conferences, Publications, and Research Projects. The average Teaching Productivity obtained by the teachers surveyed is 3.63, which can be considered intermediate since it is measured between the values “less than 2” (minimum level) and “greater than 4” (maximum level).

Regarding Research Productivity, those surveyed usually attend somewhat less than 2 Conferences (1.70), on average, annually. Likewise, teachers in the sample have participated in 8.75 Research Projects as principal investigators or team members. In addition, the participants have a total of almost 28 Publications (27.94), on average, throughout their academic life.

The standard deviation of Self-Esteem, Annual Conferences, and Teacher Evaluation is low, thus, we can affirm that most of the data collected in the sample grouped around the mean aforementioned. On the other hand, the Research Projects and, above all, the Publications, with a high standard deviation indicate a high level of dispersion in data, with little concentration around the mean. The high standard deviation in the item Annual Conferences and Publications is due to the fact that these data are not homogeneous throughout the sample, in the sense that there are teachers with very different scientific contributions in terms of Conferences and Publications, given their seniority in the job.

On the one hand, in view of the coefficient of variation, a high dispersion is observed for Research Projects (cv = 1.36), Annual Conferences (cv = 1.25), and Teacher Evaluation (cv = 1.04). On the other hand, the degree of dispersion is lower both for Self-Esteem (cv = 0.22) and for Publications (cv = 0.16).

These results are then completed with an ANOVA analysis, in order to compare the mean difference of the explanatory variable Self-Esteem according to gender, age, seniority, and professional category (Table 3).

**TABLE 3:** Comparison with the difference of means of variable Self-Esteem according to control variables.

	Mean	F welch	Sig.
**Gender**			
Male	2.704	0.50	0.48
Female	2.755102		
**Age**			
Between 30 and 40 years old	2.647727	4.129	0.00291**
Between 40 and 50 years old	2.708333		
Between 50 and 60 years old	2.894737		
More than 60 years old	2.9375		
Less than 30 years old	2.4		
**Seniority**			
Between 10 and 15 years	2.621622	2.696	0.0313*
Between 15 and 20 years	2.825		
Between 5 and 10 years	2.630137		
More than 20 years	2.90625		
Less than 5 years	2.672414		
**Professional category**			
Assistant Lecturer	2.677419	1.299	0.238
Professor	3		
Contracted Lecturer	2.7		
Interim Contracted Lecturer	2.684211		
Research Assistant	3		
Associate Lecturer	2.64		
Visiting Lecturer	2.62069		
Interim Tenured	3		
Senior Lecturer	2.825397		
Interim Senior Lecturer	3		

Significance level: *P < 0.05; **P < 0.01; ***P < 0.001.

First, we observe that there is a statistically significant difference in the means for Self-Esteem according to age (0.00291^**^) and seniority (0.0313*), since for both control variables the significance level is less than 0.05. In return, the difference in means is not significant for gender and professional category.

To carry out the analysis of the mean of the explanatory variable Self-Esteem, it is necessary to take into account that it takes values between 1 (low level) and 3 (high level), considering 2 as the average level of Self-Esteem. The results, observable in Table 3, show how Self-Esteem is slightly higher in women than in men, both being at an intermediate-high level. Taking into account the age criterion, the teachers surveyed with the highest levels of Self-Esteem are those over 60 years old, with a level very close to 3 (2.94) that can be considered to have high Self-Esteem. Teachers between 50 and 60 years old (2.89), and between 40 and 50 years old (2.71), respectively, are the next age group with the highest levels of Self-Esteem after those over 60. For their part, respondents between 30 and 40 years old (2.65) have intermediate-high Self-Esteem. Those under 30 years old are the age group with the lowest level of Self-Esteem in the entire study (2.4), which is slightly above intermediate Self-Esteem. Results show that age is an influential factor in the level of Self-Esteem since older teachers are those who show the highest levels of Self-Esteem.

Regarding seniority in the university environment, teachers with more than 20 years of experience show the highest Self-Esteem (2.91), very close to the maximum value (3). Next, from highest to lowest Self-Esteem would be those with seniority of between 15 and 20 years (2.82), those who have been in the sector for less than 5 years (2.67), those who have experience of between 5 and 10 years (2.63), and finally, those with an age of between 10 and 15 years (2.62). The latter are the ones with the lowest level of Self-Esteem, standing at an intermediate-high level. This result is consistent with the one obtained previously in relation to age since it is understood that the teachers with more seniority are also older.

Finally, considering the professional category, those who show the highest level of Self-Esteem are those who hold the positions of Professor (3), Research Assistant (3), Interim Tenured (3), and Interim Senior Lecturer (3). On the other hand, the category of Visiting Lecturer (2.62) is the one with the lowest level of Self-Esteem, followed by the profiles of Associate Lecturer (2.64) and Assistant Lecturer (2.68). Data show how the Professor category has the highest level of Self-Esteem (3), and the Visiting Lecturer (2.62) has an intermediate-high Self-Esteem, being the lowest in the sample. These results show that there is a relationship between the contractual situation of teachers and their level of Self-Esteem since it is understood that the greater job security and greater recognition of merit, the greater Self-Esteem.

In summary, significant mean differences have been identified for age and seniority. The greater age and seniority, the higher levels of Self-Esteem are observed in the sample.

Next, we proceed to analyze the relationship between Self-Esteem and Productivity, for each category of teaching staff according to their gender (Table 4).

**TABLE 4:** Comparison with the difference of means of variable Productivity according to professional category and gender.

	Self-Esteem	Teaching Productivity	Research Productivity		
		**Teacher Evaluation**	**Publications**	**Annual Conferences**	**Research Projects**
**Professor**					
Male	3	3.73	79.45	2.27	25
Female	3	3.75	126.25	4	20.25
**Interim Senior Lecturer**					
Male	3	3.5	112.5	4.5	9.5
Female	3	3	20	1	6
**Senior Lecturer**					
Male	2.81	3.69	54.19	1.44	14.91
Female	2.84	3.81	49.25	2.19	17.32
**Interim Tenured**					
Male	N/A	N/A	N/A	N/A	N/A
Female	3	3	60	1	8
**Interim Contracted Lecturer**					
Male	2.75	3.75	29	3.75	7
Female	2.67	3.67	24.8	1.8	7.67
**Contracted Lecturer**					
Male	3	3.71	23.43	1.43	9
Female	2.54	3.69	23.38	1.69	11.54
**Assistant Lecturer**					
Male	2.5	3.57	23.5	1.21	6.71
Female	2.89	3.88	11.47	1.82	4.65
**Research Assistant**					
Male	3	3.25	3.75	1	3.25
Female	3	3.75	0.75	5	1.25
**Visiting Lecturer**					
Male	2.63	3.16	2.58	0.89	3.27
Female	2.61	3.67	4.64	1.38	2.1
**Associate Lecturer**					
Male	2.5	3.5	5.97	1	2.81
Female	2.89	3.61	12.89	1.72	6.5

On the one hand, the figure of the Professor, who has the highest possible level of Self-Esteem (3) presents the highest number of Publications (126.5 for women) and Research Projects (25 for men and 20.25 for women) of all the samples. In addition, their participation in Annual Conferences is 4 for women, being one of the highest figures. Similarly, it occurs with the Teacher Evaluation, also being one of the highest in the sample (3.75 out of 5 for women). Therefore, the Teaching and Research Productivity of the Professor are both high. In view of these results, it can be seen how teachers with the highest Professional Category who have one of the highest levels of Self-Esteem show high Productivity both at the Teaching and Research levels. It should be noted that these teachers have a longer careers, so it makes sense that their Productivity is also greater.

The figure of Visiting Lecturer is the category that has the lowest levels of Self-Esteem in the sample (2.62), and that also presents low data on both Teaching and Research Productivity, comparing them with the rest of the Professional Categories. Looking at the variables, respectively, we see how Teaching Productivity (Teacher Evaluation) has one of the lowest ratings (3.16 out of 5 for women). The results are also scarce for Research Productivity, specifically for the item Publications (2.58 for men) and Research Projects (2.10) which also present the lowest numbers in the sample. In addition, analyzing attendance at Conferences annually, the minimum of the entire sample is observed in this professional category (0.89) for Female Visiting Lecturers.

The results observed for the figures of the Professor and Visiting Lecturer allow us to accept Hypothesis 1 that Self-Esteem improves the Productivity of workers since the highest levels of Productivity are observed in the category with high Self-Esteem, while the category with the lowest Self-Esteem of the sample presents low levels of Productivity.

On the other hand, it is worth noting the differences observed in the results according to gender. The greatest difference occurs in the Publications for Interim Senior Lecturers, with a difference of 92.5 articles between men (112.5) and women (20). A notable difference has also been observed in the Publications of Male Professors (79.45) and Female Professors (126.5), being greater in women with 46.8 more articles compared to men. The difference is also pronounced between the profile of Male Assistant Lecturers (23.5) and Female Assistant Lecturers (11.47). Regarding the Annual Conferences, the main difference can be seen in the figures for Interim Senior Lecturer, where men attend 4.5 Conferences, while women attend only 1, and Research Assistant, women attending 5 Conferences and men 1. In relation to Research Projects, Male Professors participated in 25, while Female Professors participated in less than 5 (20.25). For their part, Associate Lecturers present participation in Projects of 6.5, while their male counterparts have only participated in 2.81. No notable differences have been identified in the Teacher Evaluations.

The results achieved in the linear regression analysis (Table 5) can allow accepting the proposed hypothesis (significance levels lower than 0.05 for all variables), so that Self-Esteem improves the Productivity of workers within the university field of higher education, especially in the Teaching aspect.

**TABLE 5:** Linear regression analysis.

		Estimate	Std. error	*t* value	Sig.	*R* ^2^
Self-Esteem	Intercept	1.38	0.2	6.71	0.000000000112***	0.1639
Research Productivity	Annual Conferences	0.06	0.02	2.98	0.00313**	
	Research Projects	0.0083	0.0032	2.56	0.011*	
	Publications	0.0024	0.0009	2.63	0.00889**	
Teaching Productivity	Teacher Evaluation	0.37	0.05	6.56	0.000000000156***	

Significance level: *P < 0.05; **P < 0.01; ***P < 0.001.

## 5. Discussion

The results of this study show that Self-Esteem is positively correlated with Productivity, supporting what was stated by Lyubomirsky et al. (2005), Pfeffer (2018), and Zelenski et al. (2008) that happy workers are more productive and manage high job expectations more effectively than their dissatisfied counterparts.

The highest levels of Self-Esteem in the study have been identified for women over 60 years old, with more than 20 years of seniority in the university field, who hold the positions of Professor, Research Assistant, Interim Tenured, or Interim Senior Lecturer as a professional category.

However, the lowest levels of Self-Esteem are identified with the following profile: men under 30 years old, with seniority of fewer than 5 years, who hold the professional category of Visiting Lecturer or Associate Lecturer.

Results show how age is an influential factor in Self-Esteem, according to the studies carried out by Orth et al. (2015), where it is revealed how the highest levels of Self-Esteem of individuals occur around 60 years old.

In addition, the highest levels of Teaching Productivity and Research Productivity of the sample correspond to the figure of Professor, results that show what is exposed by the Human Capital Theory, which affirms that those workers who have greater labor seniority will be more productive (De Sivatte Font et al., 2018). Furthermore, a clear relationship between the Professional Category and the level of Self-Esteem is observed, understanding that the greater the security in the job position and the greater the recognition of merit, the greater the Self-Esteem will be, as defended by studies Mezzadri’s (2021).

Furthermore, the results show how Self-Esteem increases as age, professional category, and seniority in the sector increase and, on the other hand, how Productivity is higher the longer the job seniority. For this reason, we affirm that the Self-Esteem of the workers is increasing with the passing of the years, improving Labor Productivity.

The results of this study also support the conclusion that workers with a high level of Self-Esteem see themselves as capable and competent, with a valuable role they play in the organization. Self-Esteem at work is associated with commitment to the organization and performance, among other behaviors and attitudes linked to the organization, as shown by studies by Pierce et al. (1989), Pierce and Gardner (2004), Kim and Beehr (2018), Neves et al. (2020), and Rice et al. (2020).

Large articles highlight that Self-Esteem helps employees improve their performance by helping them to deal with job stress, anxiety, and depression. Therefore, the results of the study (the figure of the Professor who holds the highest level of Self-esteem possible presents a high Productivity, both Teaching and Researcher) reinforce the idea that behavior and performance are related to the Productivity of employees and have the potential impact on their career advancement (Tharenou, 1979; Gardner et al., 2004; Brough et al., 2009; Gordon and Hood, 2020; Kim et al., 2021).

Throughout the analysis, the relationship between Self-Esteem and the control variables is observed: age, seniority, and professional category have a great influence on the level of Self-Esteem not only individually but also acting all three together.

With respect to gender, marked differences in Research Productivity are observed for Publications (Professor and Interim Senior Lecturer), Annual Conferences (Interim Senior Lecturer and Research Assistant), and Research Projects (Professor).

It is relevant to consider the idea that the organizational climate predicts organizational commitment, work performance, employee morale, and the behavior of workers in the organization, and assuming an increase in the level of Self-Esteem in the long term, the importance for universities to ensure a cultural organizational climate that contributes to the development of teachers’ Self-Esteem, influencing their Teaching and Research Productivity. The reduction of the workload and stress factors, the increase in the complexity and responsibility of work, success in the position, support for workers, and their empowerment, are some of the variables that help to improve efficiency and Self-Esteem in the work environment and that could be used by universities, having an impact on their workers, students, and the institution itself.

## 6. Conclusion

The results obtained show the increase in Self-Esteem of teachers over the years, improving their labor productivity, and the importance of suggesting proposals that promote Self-Esteem in the teaching field, given its impact on teachers, the university, and society, being able to extrapolate these results to other organizations.

Thus, the objective of the study has been achieved, showing the relationships between the Self-Esteem of teachers in the field of higher education in Spain and their Teaching and Research Productivity. Specifically, the relationships established between Self-Esteem and the Evaluation received by teachers from their students, between Self-Esteem and Publications, between Self-Esteem and attendance at Conferences annually, and between Self-Esteem and participation in Research Projects are identified. In addition, the levels of Self-Esteem are evaluated according to the control variables: gender, age, seniority in the university field, and professional category. The results of the study indicate that analyzing Self-Esteem in the workplace could be very necessary for understanding the underline patterns of individual issues of teachers to increase their Productivity.

### 6.1. Theoretical and practical contributions

This article has important contributions. From the theoretical point of view, we clarify what has been studied in the literature about Self-Esteem and Productivity, which may be useful for other researchers, developing a theoretical model that brings together the main relationships of Self-Esteem in the work environment with key aspects of the worker, specifically their Productivity. From a practical point of view, the results reveal the incidence of Self-Esteem in Teacher Evaluation, Publications, attendance at Conferences, and participation in Research Projects, i.e., the influence of teachers’ Self-Esteem on their Teaching and Research Productivity. These results suggest the importance of universities ensuring the Self-Esteem of their workers, enhancing it would improve the behavior of teachers in terms of Productivity, having a very positive impact on the education received by students and on the contribution to specialized literature. The implementation of practices that foster teachers’ Self-Esteem would have an impact on the teaching center and its prestige, on the teachers themselves and their students, and in society, increasing academic research and educational quality. Similarly, the results achieved can be extrapolated to other sectors.

### 6.2. Limitations and future lines of research

The limitations of the article try to be overcome by suggesting different lines of future research.

First, one of the limitations found in the study is associated with the reliability of the information provided by the respondents since the method used to collect the information has been the survey. This instrument allows for obtaining the information that was necessary to measure the variables of the model and to access the targeted population in a reasonable period of time. However, the use of this technique means accepting certain inherent drawbacks, such as the low response rate associated with it or the loss of possible information that could be obtained with other methods.

Second, the study is carried out only in one university, the sample not too large, assuming that the results obtained cannot be generalized, so in the future, it is proposed to extend the research to other universities, in order to have a larger number of observations. It is also proposed to extend the research to other fields of study.

In addition, the article is limited to analyzing the relationship between Self-Esteem and Teaching and Research Productivity, and the analysis of other variables such as motivation, job satisfaction, or altruism of teachers may be very relevant, allowing us to know the impact of Self-Esteem on more variables in the teacher’s work environment.

In the end, we consider that the approach of the contribution of Self-Esteem on sustainability and the scope of the Sustainable Development Goals (SDG) could be of vital interest to contribute positively to the fulfillment not only SDG 3 (Good Health and Wellbeing) and SDG 4 (Quality Education) but also on SDG 8 (Decent Work and Economic Growth) in case it is extrapolated to other sectors. Similarly, it is suggested the approach of a protocol of action by the university that promotes Self-Esteem among teachers in order to improve their Productivity.

## Data availability statement

The raw data supporting the conclusions of this article will be made available by the authors, without undue reservation.

## Author contributions

Both authors participated in the writing and revisions of the manuscript, have made a substantial and intellectual contribution to the work, and approved it for publication.
