# Supplementary material for: The relation between Self-Esteem and Productivity: An analysis in higher education institutions
Source: Front Psychol. 2023 Jan 11;13:1112437. doi: 10.3389/fpsyg.2022.1112437 (PMC9875080; doi:10.3389/fpsyg.2022.1112437)
Supplement: Supplementary file 1 [file Data_Sheet_1.docx]

**ANNEX**

**Annex 1. Self-Esteem variable measurement. Rosenberg scale (1965) adapted to the work environment.**

Items used:

| 1 | I feel that I am a person worthy of appreciation in my job (at least to the same extent as my colleagues) |
| --- | --- |
| 2 | I am convinced that I have good qualities to perform my job |
| 3 | I am able to do things as well as the rest of my colleagues |
| 4 | I have a positive attitude towards myself in my workplace |
| 5 | In general, I am satisfied with myself in my job |
| 6 | I feel like I don't have much to be proud of in my workplace |
| 7 | In general, I think that I am a failure in the work context |
| 8 | I would like to be able to feel more respect for what I do in my job |
| 9 | There are times when I really think that I am useless in my job position |
| 10 | Sometimes I think I'm not a good person |

Response scale used:

| 1. Strongly disagree | 2. Disagree | 3. Neither agree nor disagree | 4. Agree | 5. Strongly agree |
| --- | --- | --- | --- | --- |

**Annex 2. Productivity variable measurement. Based on National Agency for Quality Assessment and Accreditation (ANECA) requirements.**

Teaching Evaluation

| Indicate the results (on average) that you usually obtain in Teaching Evaluation surveys (with 1 being the minimum and 5 the maximum). |
| --- |

Response scale: Less than 2 / Between 2 and 3 / Between 3 and 4 / Greater than 4

Publications

| Indicate the number of publications made throughout your career |
| --- |

Annual Conferences

| Indicate the number of conferences you usually attend annually |
| --- |

Research Projects

| Indicate the number of research projects and/or contracts in which you have participated throughout your career |
| --- |
